# *Microchloropsis gaditana* as a Natural Antimicrobial with a One Health Approach to Food Safety in Farmed Salmon

**DOI:** 10.3390/life15030455

**Published:** 2025-03-13

**Authors:** Nelson Díaz, Susana Muñoz, Alberto Medina, Carlos Riquelme, Ivonne Lozano-Muñoz

**Affiliations:** 1 Departamento de Producción Animal, Facultad de Ciencias Agronómicas, Universidad de Chile, Santa Rosa 11315, Santiago 8820000, Chile; ndiaz@uchile.cl (N.D.); smunoz@uchile.cl (S.M.); 2 Departamento de Acuicultura y Recursos Agroalimentarios, Universidad de los Lagos, Alberto Hertha Fuchslocher 1305, Osorno 5380000, Chile; amedina@ulagos.cl; 3 Centro de Bioinnovación, Facultad de Ciencias del Mar y Recursos Biológicos, Universidad de Antofagasta, Angamos 601, Antofagasta 1270300, Chile; criquelme@uantof.cl

**Keywords:** food safety, *Microchloropsis gaditana*, farmed salmon, microalgae, natural antimicrobials, one health

## Abstract

Sustainably farmed Atlantic salmon could drive global food system solutions by contributing essential nutrients to the human diet while delivering high-quality protein. One of the biggest obstacles to sustainable salmon aquaculture in Chile is the prevalence of piscirickettsiosis disease caused by the Gram-negative bacteria *Piscirickettsia salmonis* and the excessive amount of antibiotics used to eradicate this disease. Farmed salmon products can be consumed without prior processing and therefore present a substantial risk for the transfer of resistant pathogens to humans. Antibiotics also carry the risk of antibiotic residues and damage to the environment. An alternative to antibiotics is the use of natural antimicrobials without the negative influence on the consumer’s microbiome. Here, we evaluate the potential antimicrobial activity against *P. salmonis* of the marine microalgae *Microchloropsis gaditana*. A non-genetically modified *M. gaditana* was grown with nitrogen deprivation to improve the synthesis of the eicosapentaenoic fatty acid (EPA). A spray-dried *M. gaditana* concentrate (Mg) was elaborated and given to Atlantic salmon for a period of 49 days, and serum and fillet samples were collected. Our results showed a significant increase in the nutritional quality improving the levels of EPA+ Docosapentaenoic acid (DPA) (23%) and Vitamin D_3_ (106%) of the fillets treated with Mg. Fish fed serum were challenged with *P. salmonis*, and serum antibacterial activity was measured. Sera from fish fed Mg-enriched diets showed a significant increase in antibacterial activity (85.68%) against *P. salmonis*. Our results indicate that Mg can be used as a viable alternative to address the critical problem of microbial resistance and to assure consumers that farm-raised Atlantic salmon is safe.

## 1. Introduction

Despite efforts to reduce the use of antimicrobials in salmon farming in Chile, its use increased by 18% in 2021 (463.4 tons) versus 2020 (379.6 tons). The total production of salmonids decreased by 8.35%. Of the total amount of antimicrobials used in 2021, 98.74% were administered in the marine stage (Patagonian fjords and channels), and 97.1% was Florfenicol used in Atlantic salmon (92.83%) to control piscirickettsiosis (93.17%) [1]. In 2023, this disease was responsible for 44.7% of infectious deaths in Atlantic salmon, being the most important infectious cause in this species [2]. The increased overuse of antibiotics suggests that measures used to reduce their use have failed. Antibiotic residues as well as antimicrobial-resistant bacteria from salmon production are spreading in the environment [3,4,5,6], and both wild organisms and salmon food commodities can become a source of resistant bacteria that can be transmitted to humans as foodborne contaminants [6,7,8,9]. There is thus a need for new and sustainable strategies. Antimicrobials originating from natural sources are not only safe but constitute a viable alternative to solve the problem of microbial resistance while meeting the requirement for healthy foods [10].

*Piscirickettsia salmonis* is the causative agent of SRS (Salmonid Rickettsial Septicemia) [11]. Salmon affected by *P. salmonis* respond inconsistently or poorly to treatment with Florfenicol [12,13,14,15], and several studies have reported that *P. salmonis* developed resistance to antibiotics [13,16] that can generate new antimicrobial resistance among bacteria, with negative effects on aquaculture and human health [17]. The evolution of resistance of *P. salmonis* to antibiotics has been demonstrated: A large-scale study evaluated the susceptibility profiles for Oxytetracycline and Florfenicol from 292 field isolates obtained from different farm sites over a five-year period. The results revealed resistance to Florfenicol and Oxytetracycline [18,19].

There is emerging evidence that adequate nutritional support can increase the ability of fish to resist infectious diseases by maintaining intestinal health and thus increasing immunity. Dietary components affect intestinal health through several pathways including intestinal barriers, digestive enzyme activity, oxidative status, and microbial diversity that are essential in controlling general fish immunity [20]. The essential fatty acids eicosapentaenoic acid (EPA) and docosahexaenoic acid (DHA) have an important anti-inflammatory role in immune regulation during infections in Atlantic salmon [21,22]. The reported dietary requirement of the n-3 fatty acids EPA, alpha linolenic acid (ALA), and DHA of salmonids are 10–25 g/kg feed. Atlantic salmon fed low ratios of n-3/n-6 PUFAs (Polyunsaturated Fatty Acids) are more susceptible to infections [23]. Fish fed manufactured diets with an increasing content of plant-based products have shown metabolic disturbances with a low PUFA content in their muscles related to the composition of vegetable oils. The essential fatty acid requirements of freshwater fish can be met by adding linolenic acid (18:3n3) or linoleic acid (18:2n6), but EPA and DHA are regarded as essential fatty acids in marine fish due to the inability of all marine fish to convert 18:3n3 to EPA and DHA as well as 18:2n6 to arachidonic acid (ARA 20:4n6) [24].

The ability of Atlantic salmon to osmoregulate is directly related to the diet and is mediated through changes in tissue polar lipid fatty acid composition. Elevation of omega-3 PUFAs is an indicator of a pre-adapted fish for entry into seawater [22]. Changes in the composition of PUFAs have been shown to be involved in adaptation to seawater and have affected the production of prostaglandins that mediate the flow of electrolytes and fluids and operate synergistically with the prolactin hormone in the regulation of plasma Na^+^ and Ca^+2^ concentrations [22,25]. EPA and DHA deficiencies in the early life stages of fish impact their ability to deal with challenging conditions [26]. Non-GMO (genetically modified organism) terrestrial plant ingredients used for the formulation of fish diets affect the composition of deposited fatty acids by decreasing the concentration of EPA and DHA in turn reducing the resistance of fish to diseases and presenting physiological alterations that affect their performance. This process can also lead to lower levels of EPA and DHA in the final seafood; levels sometimes do not cover the minimum required levels of these fatty acids [27,28].

Microalgae in their natural ecosystem are producers of bioactive compounds with antimicrobial properties. Many marine algae derivatives are promising novel antimicrobial agents with multiple applications, including in the food and pharmaceutical industries [10]. Microalgae are producers of PUFAs that are subsequently consumed by other aquatic organisms and contribute to the healthy fatty acid profile in fish [28,29,30]. Microalgae are rich in oil and mineral content and can reproduce their biomass (1–3 per day). This is 50-fold higher than other terrestrial plants [31]. The microalgae *Microchloropsis* sp. is widely distributed in oceans worldwide [31] and is well known to produce PUFAs. It can naturally produce high levels of vitamin D_3_ [32]. Stress parameters (temperature, nutrients, light intensity) are known to improve the synthesis of PUFAs rich in EPA in *Microchloropsis* sp. [29,33], along with reasonable amounts of ARA and significant amounts of minerals. The mineral content of the biomass has been published: (mg/100 g) of magnesium (1490.1–3039.6), calcium (751.68–2338.8), potassium (190.74–1424.4), iron (91.68–214.32), zinc (23.4–52.68), manganese (11.52–75.12), copper (7.44–14.64), and nickel (3.96–4.68) [33]. Nitrogen limitations are considered the most efficient strategy to increase the content of triglycerides composed of fatty acids with a high degree of saturation in microalgae [34].

Fish do not synthesize vitamin D_3_ and are fully dependent on dietary sources to meet their requirements [35]. Calcium (Ca^+2^) concentrations in water regulate vitamin D_3_ in Atlantic salmon during the smoltification process and their migration from freshwater to seawater [36]. The values of vitamin D_3_ metabolites in plasma depend on the environmental concentrations of calcium present in both saltwater and freshwater. Lower environmental calcium concentrations induce higher conversion to a 1,25-dihydroxycholecalciferol-like compound but increased environmental calcium is associated with higher transformation to the compound 25,26-dihydroxycholecalciferol [36]. Vitamin D is known to regulate calcium homeostasis [37] and is a promising candidate to combat infections caused by bacteria (including multi-drug resistance). It exerts fundamental functions in innate immunity with broad antimicrobial effects. Vitamin D receptors are expressed on innate and adaptive immune cells. The 1,25(OH)2-vitamin D-active form of vitamin D has been shown to increase cathelicidin antimicrobial peptide gene expression in innate immune cells such as macrophages and monocytes [38,39]. The elimination of pathogens by cathelicidin may partially explain the antimicrobial properties of vitamin D [38].

The aim of this study was to evaluate the effect of two different levels of *Microchloropsis gaditana* (1.0 and 10.0%) with high levels of EPA as a functional anti-*P. salmonis* LF-89 additive to be used in salmonid diets as a sustainable nutritional strategy alternative to the use of antimicrobials—specifically Florfenicol—for the control of *P. salmonis* strains.

## 2. Methods

### 2.1. M. gaditana Growth Conditions, Concentrate, and Characterization

The *M. gaditana* strain (Lubián CCMP 527) was maintained in 250 mL flasks at 20 °C under constant illumination at 200 μE m^−2^ s^−1^ via a fluorescent lamp under UMA 5 nitrogen and phosphorous deprivation culture medium (NaNO_3_: 0.4 g/L, NaHCO_3_: 0.168 g/L, NaH_2_PO_4_: 0.034 g/L, and trace elements μmol/L, Mn 0.90, Zn 0.08, Co 0.050, Mo 0.030, and Fe 11.70, Centro de Bioinnovación, Antofagasta, Chile). The culture medium was autoclaved for 15 min at 121 °C and prepared using natural seawater with analytical-grade nutrients [34]. The *M. gaditana* strain was sub-cultured every ten days by adding 10% of the culture media to 90% of fresh culture medium. The lack of contamination was monitored by microscopic observations (Leica CME microscope 40×/0.65, Leica, Wetzlar, Germany). The culture was scaled to 1 L (under the same conditions) and then to an open raceway system (14.4 m^3^) under outdoor conditions. It was then grown in batch mode and maintained for 15 days. Cultures were hatched at 20 °C and initiated in a semi-continuous mode in which 75% of the culture medium and vitamin supplementation were deprived.

The *M. gaditana* was subsequently harvested (December 2018) at a dilution rate of 10% per day. Semi-continuous culture in outdoor conditions allowed to reach 2.6 g EPA/100 g biomass and volumetric productivity of 46–56 mg/L/day to be reached. Microalgal cells were harvested by continuous centrifugation (GEA Westfalia, model AS 1936076, Oelde, Germany) at a flow rate of 2 m^3^/h and a pressure of 3 bars. After centrifugation, the Mg was concentrated and dehydrated by spray drying (LPG-25 high-speed centrifugal spray dryer, Changzhou Yibu Drying Equipment Co., Ltd., Zengzhou, Henan, China) with a flow rate of 4 L/h, an initial inlet air temperature of 185 °C and a drying chamber temperature of 90 °C with a final temperature of 80 °C/15 s. Fatty acid profiles, minerals, and proximate analyses in Mg concentrate were determined using the official methods of analysis of the Association of Official Analytical Chemists (AOAC): crude fat [40,41]; fatty acid profile [42]; protein [43] using the calculation 5.25XN value; ash determination [44], moisture content [45]; and vitamin D_3_ [46] with a limit of quantification (LOQ) of 0.25 μg/100 g.

### 2.2. Experimental Diets and Fish

Three diets were manufactured into 3 mm diameter extruded pellets using a Clextral BC-21 (Firminy, France). These included a control diet and two diets with Mg inclusion (1 and 10%). After extrusion, pellets were dried at 60 °C for 12 h. In those with Mg supplementation, the Mg was dissolved in fish oil and added to the extruded basal feed. The oil was incorporated according to each diet formulation using a Dinnissen VC10 vacuum oiler (Sevenum, The Netherlands). Diets were prepared in batches of 10 kg. The formulations and approximate composition of the experimental diets are shown in Table 1.

Atlantic salmon (*n* = 225; 104 ± 1.29 g), each from AquaGen single family (SNAQ16LSSCO), were randomly distributed in nine tanks (three tanks per diet). Prior to the start of the trial, the fish (undifferentiated sex: in typical Atlantic salmon cycle schemes, sexual maturation is reached after a period of growth in the sea, generally 1 to 4 years) were acclimatized for 15 days. Fish were fed one of three diets for 49 days (March–May 2018) at Piscicultura Iculpe-Ilihue, Lago Ranco, Chile experimental station. The fish were maintained at 8.51 ± 0.14 dissolved oxygen concentration, pH 7.11 ± 0.04, 8.6 ± 1 °C, 24 h light photoperiod and were held in 200 L tanks with fresh water supply (flow-through system). The study was approved by the Bioethics and Biosafety Committee of the Faculty of Agronomic Sciences University of Chile Nº74-2018. This study was reported following the ARRIVE guidelines [47].

The fish were fed to satiation twice a day. Feed consumption, dissolved oxygen concentration, temperature, and pH were measured daily. Fish weight and length were recorded at the beginning and at the end of the trial. Indices of fish growth and feed utilization were calculated as follows:

- Weight gain (%) = 100 [(W2 − W1)/W1]
  where W2 = final weight and W1 = initial weight of fish
- Specific growth rate (SGR, %/day) =100 × (ln W_2_ − ln W_1_)/feeding days where ln is the natural logarithm, W_1_ = initial weight and W_2_ = final weight of fish.
- Feed conversion ratio (FCR) = feed consumed/ biomass increase.
- Survival rate (SR%) = (final fish number/initial fish number) × 100.
- Feed intake (g/day/individual) = total feed intake/(days × number of fish)
- The Fulton’s condition factor (*K*) of the experimental fish was calculated as: *K* = 100 W/L^3^ [48] where W = weight of the fish in grams and L = total length of the fish in centimeters.

### 2.3. Serum and Fish Fillet Sampling

For antibacterial activity, ten fish per tank for blood samples were taken after a 12 h fast on days 0 and 49. For blood extraction, to reduce movement the fish were placed on an ice gel pack for approximately 30 s, and a 2 mL sterile syringe was used to extract 1.0 mL of blood from the caudal vein, which was collected in a 2.0 mL cryo vial. The fish were returned to the tank for recovery, which took about 1 min. Serum was obtained from blood by low-speed centrifugation (3000 rpm for 10 min at 4 °C) within 1 h after sampling. Serum samples were immediately placed in liquid nitrogen and subsequently stored at −80 °C.

For fatty acid profiles, vitamin D_3_, ash, and proximate analyses, three fish per tank were randomly sampled on day 49. All animals were fasted for 12 h before sampling. The fish were anesthetized with MS-22 (80 mg/L/4 min, Argent Chemical Laboratories Inc., Redmond, WA, USA) [49] before being slaughtered by cervical dislocation according to Directive 210/63/EU [50]. Skin, liver, and gut were removed and the resulting fillets were lyophilized (FDT 8632 model freeze dryer) and stored for further analysis. All procedures including treatment, euthanasia, and handling were performed according to the guidelines of the University of Chile animal welfare committee. Fatty acid profiles, minerals, and proximate analyses in fish fillets were determined using the official methods of analysis of the AOAC: crude fat [51]; fatty acid profile (Chemists AOAC 2012); protein [43] using the calculation 6.25XN value; ash determination [52], moisture content [45]; and vitamin D_3_ [46] with a limit of quantification (LOQ) of 0.25 μg/100 g.

### 2.4. Anti-Piscirickettsia salmonis Assay

The strain *P. salmonis* ATCC VR-1361, equivalent to LF-89, was used from the American Type Culture Collection (ATCC, Manassas, VA, USA). The bacterial strain was confirmed as *P. salmonis* using an indirect fluorescent antibody test (IFAT, SRS-Bios, Santiago, Chile) according to the manufacturer’s recommendation. The strains were cultivated in the Austral-TSFe agar plates and incubated at 18 °C for 8 to 10 days. Stock cultures were prepared from cells scraped off TSFe plates and resuspended in L-15 Lebovitz medium (1 mL) with 20% of fetal bovine serum (Biological Industries Ltd., Israel Beit Haemek, Israel) and 10% of dimethyl sulfoxide (Sigma-Aldrich, ≥99.7%, St. Louis, MO, USA) and stored at −80 °C. Colonies grown on Austral-TSHem plates were used to prepare the inocula of *P. salmonis* in sterile Austral-SRS broth [53,54]. Purity controls were used in each of the methodological steps via Gram staining. The concentration of the initial inoculum was quantified by microscopic counting and microbiological seeding while recording the number of colony-forming units (CFU/mL) in Austral-TSHem plates [54]. The cultures were diluted to an optical density (OD) of 600 nm = 0.1 and returned to the exponential growth phase (OD 600 nm = 0.18–0.2) prior to use in anti-*P. salmonis* assays.

The bacteria (10 μL) in exponential growth culture and 50 μL of each serum were added into a 96-well plate (NEST^®^, Xuzhou, Jiangsu, China) in triplicate. Negative controls had 50 μL of serum and 10 μL of bovine fetal serum (Biological Industries, Israel Beit Haemek Ltd., Israel Beit Haemek, Israel). Positive controls had 10 μL of the bacteria in 50 μL of nutrient broth.

The anti-*P. salmonis* activity in fish fed serum was measured using an MTS (3-(4,5-dimethylthiazol-2-yl)-5-(3-carboxymethoxyphenyl)-2-(4-sulfophenyl)-2*H*-tetrazolium) assay based on the reduction of MTS tetrazolium salt to a red formazan product by dehydrogenase enzymes from live cells [55]. The assay was performed by adding 20 μL of the cellTiter 96^®^AQ_ueous_ one solution reagent (Promega, Alexandria, NSW, Australia) directly to each culture well and incubating for 2 h, followed by recording the absorbance at 490 nm with a 96-well plate reader. The quantity of formazan product was measured via the amount of 490 nm absorbance with a TECAN INFINITE^®^ 200 PRO spectrophotometer (Tecan Trading AG, Männedorf, Switzerland). The antibacterial activity of the sera (%) was calculated using the formula:

Antibacterial activity (%) = 100 − (treatment absorbance − negative control absorbance)/(positive control absorbance × 100).

### 2.5. Statistical Analysis

To test for differences between dietary treatments on *P. salmonis* antibacterial activity, data were subjected to a univariate analysis of variance. Fish growth performance indices and fatty acid retention data were subjected to a one-way analysis of variance (ANOVA) using IBM SPSS Statistics (version 25.0, IBM Corporation, Armonk, NY, USA). Multiple comparisons were made using Dunnett’s post hoc test when significant differences were identified between groups. Significant differences were considered when *p* < 0.05.

## 3. Results

### 3.1. Mg Concentrate Characterization

Nutritional characterization of the Mg concentrate showed high protein, ash, and calcium content (Table 2). Analysis of the fatty acid composition in Mg concentrate showed low levels of vitamin D_3_ and DHA. The EPA levels reached 26.73% of total fatty acids.

### 3.2. Fish Growth Performance Indices

No toxic or pathological signs were observed; experimental diets were received well by the animals. Consumption of the diet supplemented with Mg (10%) resulted in a significant increase in weight gain and the specific growth rate at the end of the 49-day feeding study. No significant differences were observed in the condition factor and feed conversion ratio (Table 3). No exclusions were made in the data analysis.

### 3.3. Salmon Fillets Fatty Acid Retention

**Figure 1:**
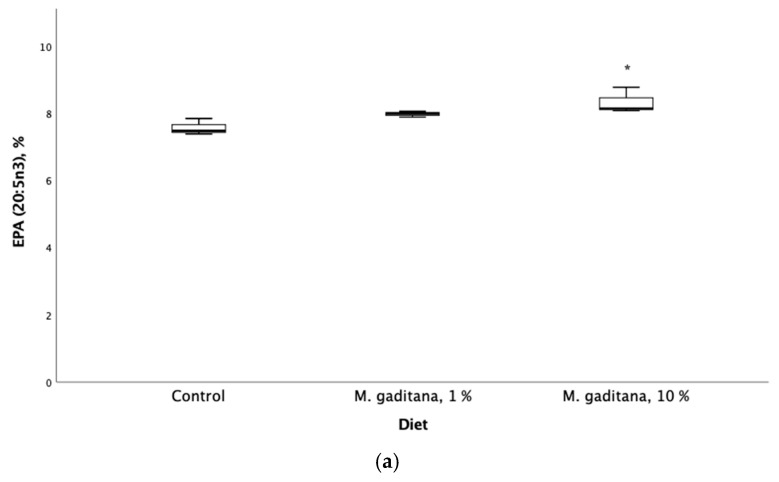

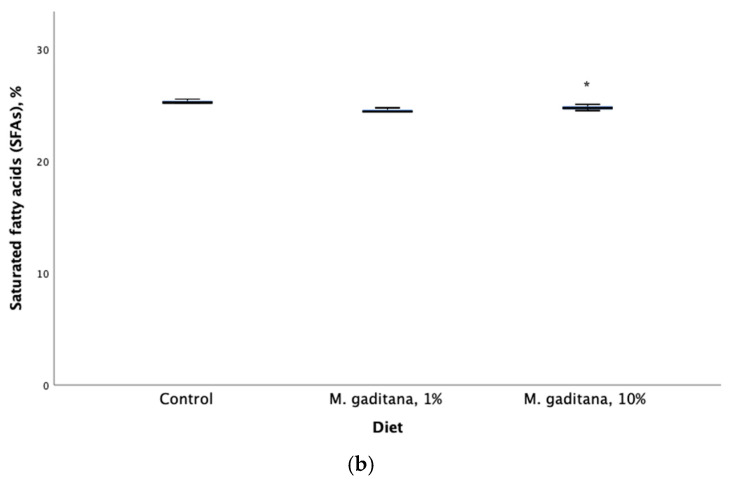
Eicosapentaenoic acid (EPA) expressed as percent of total fatty acids (**a**) and saturated fatty acids (**b**) retention in *Salmo salar* fed on *M. gaditana* concentrate (Mg)-enriched diets for 49 days. * Significant difference from the control, *p* < 0.05, *n* = 3.

The inclusion of Mg in the diet had a significant and positive effect on the retention efficiency of the anti-inflammatory fatty acids EPA (20:5n3) and DPA (22:5n3). The results showed increased levels in the n-3 family (EPA and DPA) and decreased levels of n-6 family fatty acids, including a decrease in the oleic acid pathway in fish fed on Mg-enriched diets (Table 4). The ARA/EPA ratio was significantly decreased (Figure 1).

### 3.4. Effect of M. gaditana in Anti-P. salmonis Assay

**Figure 2:**
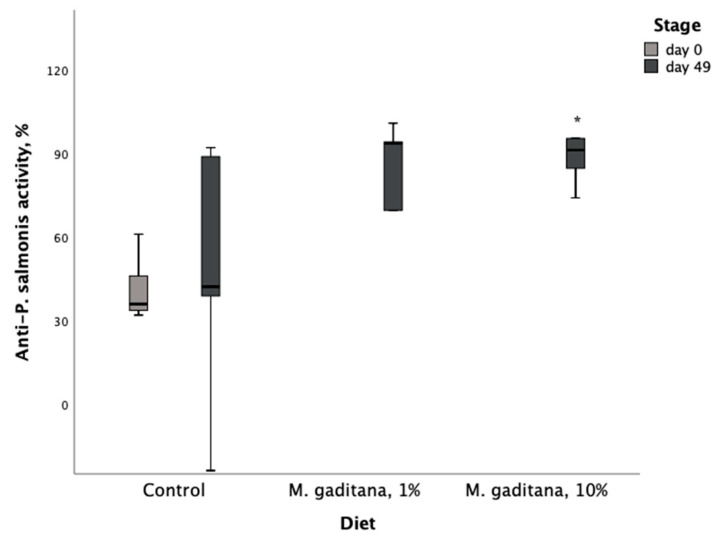
Anti-*P. salmonis* (LF-89) activity (%) in serum of *S. salar* fed on *M. gaditana* (Mg)-enriched diets for 49 days. * Significant difference from the control, *p* < 0.05, *n* = 10.

After performing the ex vivo assay, we next completed a *P. salmonis* challenge protocol. Positive and negative controls showed the expected results. Bacterial counts detected average concentrations of 4.5 × 10^8^ bacteria/mL and 4 × 10^5^ CFU/mL. Serum from fish fed the Mg-enriched diets showed higher anti-*P. salmonis* activity (Table 5). Sera from *S. salar* fed the diet with a 10% Mg inclusion level had a significant increase in antibacterial activity (%) from the control diet at 49 days of feeding (Figure 2).

## 4. Discussion

Our results showed that *M. gaditana* grown with nitrogen deprivation culture conditions is an important source of polyunsaturated anti-inflammatory fatty acid EPA (26.73%), sodium (5.40%), ash (21.26%), and calcium (4.11%). These results are consistent with EPA values reported for dehydrated samples by centrifugal spray-drying *Nannochlorpsis oceanica* (28.9%) [56] and ash content (24.47%) reported for *Nannochloropsis oculata* [31]. The *N. gaditana* can accumulate large amounts of EPA during nitrogen starvation. With controlled photoperiod conditions, EPA values of up to 37% have been reported for *N. gaditana* [33]. The resulting fish performance data from our study indicate that the inclusion of Mg-concentrate up to 10% in the diet of *S. salar* has no negative effect on performance parameters. These results agree with a previous study where the incorporation of 10% *N. oculata* in a commercial Atlantic salmon diet did not affect feed utilization and growth [57]. Another study achieved a 31% greater weight gain in Atlantic salmon when fed a diet containing 5% *Schizochytrium* sp. [58]. However, our fish performance data do not agree with a previous report where the use of *N. oceanica* with a 30% inclusion and a 60-day feeding generated significantly lower weight gains and a specific growth rate in Atlantic salmon [59]. This may be due to the inclusion percentage, differences in the nutritional content of the microalgae used in this study, or variations in microalgae processing during the preparation of the diets.

Our study showed a significant increase in the essential nutrient vitamin D_3_, which increased by 100% in fish fed Mg-concentrate relative to fish fed a control diet. This may be due to the presence of calcium (Ca^+2^) and its effects on plasma D-vitamin metabolites values, which have been reported to be dependent on ambient calcium concentrations in water in Atlantic salmon [36]. Previous studies have shown that *N. oceanica* can naturally produce high levels of vitamin D_3_ (up to 1 ± 0.3 μg/g DM) by exposure to ultraviolet-B light [32]. The potential role of vitamin D_3_ in inducing innate immunity and improving resistance against different pathogens is well-documented [60,61,62]. Vitamin D_3_ modulates the immune response of the infected host by promoting an autolysosome function [60], producing antimicrobial peptides, and inducing cell-specific receptors related to the elimination of pathogens. Vitamin D_3_ has a direct influence on macrophages and assists neutrophil motility and phagocytic function [61]. There is also clear evidence that D_3_ positively regulates the innate and adaptive immunity in fish [62].

*Nannochloropsis* sp. is a source of high-value essential n-3 long-chain PUFAs such as EPA and alpha linolenic acid [59,63]. Our results for ARA and EPA fatty acids are consistent with values previously reported when comparing *Microchloropsis*-treated fish with the control diet [59]. There was an increase in EPA content and a decrease in ARA content resulting in a significant decrease in the ARA/EPA ratio. Values of up to 35% lower in DHA (7.64 ± 0.53) and EPA (5.83 ± 0.85) levels have been reported for Chilean salmon fillets compared to those obtained in our study [64]. It has been reported that the fatty acid content of total lipid extract from the green microalgae *Coelastrella* sp. showed therapeutic potential against Gram-negative bacteria [65]. In addition, the bactericidal activity of C20 series fatty acid has shown increased potency with the increase in unsaturation, reaching maxima for EPA [66].

Compared with the fish fed a control diet, fish fed a 10% inclusion of Mg-concentrate diet had total saturated fatty acids (SFAs) that were significantly decreased (2%). Our results agree with a previous study on the muscle tissues of Nile tilapia (*O. niloticus*) fed diets supplemented with 0.75% of microalgae mix containing *Nannochloropsis oculate*, *Schizochytrium,* and *Spirulina* species in equal proportions (1:1:1). The study reported a decrease of 2.49% in SFAs, and showed that the DHA and EPA components of the microalgae mixture used have antibacterial properties against *Aeromonas hydrophila* [67].

Diets including 10% Mg-concentrate showed significant increases in antibacterial activity against *P. salmonis* relative to the control diet. Marine green microalgae antibacterial activity has been described previously against *Staphylococcus* sp., *Vibrio angullarum*, *A. hydrophila*, *Lactobacillus* sp., and *Aeromonas salmonicida* [68]. In addition, various studies have already shown that *Chlorella* sp., *Tetraselmis* sp., *Navicula* sp., *Phaeodactylum tricornutum*, *Porphyridium cruentum*, *Microchloropsis gaditana*, *Dunaliella salina*, *Lobosphaera* sp., and *Schizochytrium* sp. improve immunostimulatory capacities, resistance to infectious diseases, and tolerance to environmental stress in fish [69]. Phenolic compounds obtained from microalgae have shown inhibitory and antimicrobial effects on bacterial growth, depending on their structural properties and their amount in the environment. Studies conducted with *N. oceania* show that water-soluble polysaccharides produced by this organism can be used as antimicrobial agents [70] and that fatty acids present in microalgae *N. oculata* ably inhibit the growth of bacteria [71].

Our results indicate that *M. gaditana* grown under controlled conditions are suitable for sustainable nutrition against *P. salmonis* in Atlantic salmon due to the presence of essential nutrients that play important roles in the immune system of fish. DHA and EPA are important components of plasma membranes that are integral in controlling membrane-signaling pathways [72] and are precursors for anti-inflammatory lipid mediators [73,74] that modulate the duration and intensity of inflammatory responses in humans and fish [72]. Dietary fat is also linked to inflammation by promoting the translocation of gut microbiome metabolites from the gut into the bloodstream [73].

Lipopolysaccharide (LPS) is often described as an endotoxin and is an important virulence factor. It represents 75% of the total surface area of Gram-negative bacteria [75]. LPS is found in their outer membrane where their hydrophobic structures are composed of fatty acids. LPS plays a crucial role in bacteria–host interactions by modulating host immune system responses [73,75]. In salmon macrophage-like cells infected with *P. salmonis* LF-89, LPS has been identified to vary between stages of infection. In the vacuolization stage, proteins belonging to the “endotoxin” virulence factor family are abundant, suggesting the bacteria modifies or overproduce surface components, including LPS, allowing survival inside the host [76]. High-density lipoprotein-associated LPS favors its elimination through the liver and bile, thus preventing LPS-induced toxicity [75,77]. In the case of low high-density lipoprotein levels, most of the LPS is associated with very low-density lipoproteins with a lower neutralizing capacity [75].

**Figure 3:**
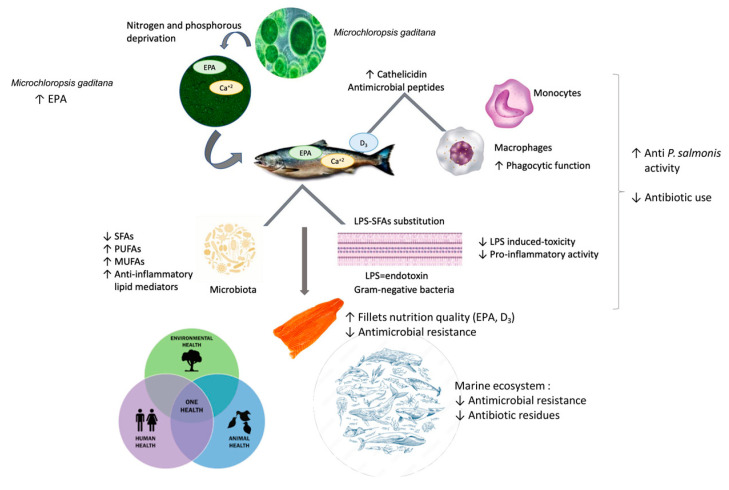
*Microchloropsis gaditana* as a natural antimicrobial with a One Health approach to food safety in farmed salmon. Synergistic effect between EPA, vitamin D_3_, and the substitution of saturated fatty acids in the anti-*P salmonis* activity.

The acute postprandial inflammatory response associated with fat consumption is mediated by endotoxins. These are mainly derived from the intestinal microflora [73]. In addition, gut dysbiosis induces gut permeability defects, leading to spontaneous endotoxemia [78]. In the case of *P. salmonis*, previous studies have apparently concluded that is an opportunistic environmental pathogen with low levels of pathogenicity and virulence and an endogenous pathobiont colonizing the salmonid microbiome [17]. SFAs induce inflammation in part by mimicking the actions of LPS. They are an essential structural component of bacterial endotoxins associated with the toxicity caused by these molecules. The substitution of SFAs with MUFAs (Monounsaturated fatty acids) or PUFAs eliminates the pro-inflammatory activity of LPS [73]. Macrophages are considered the main effectors in the defense against bacterial infection capable of responding to interactions with Gram-negative cells. Macrophages recognize these organisms in the absence of specific immune system components, and there are many effects of Gram-negative bacteria on macrophages that are mediated by bacterial lipopolysaccharides and their biologically active lipid fraction [79]. SFAs are also characterized by their role in inflammatory responses in macrophages and can induce inflammation, either extracellularly via Toll-like receptors or intracellularly via products of SFA metabolism [80]. Our results show a significant decrease in SFAs and a significant increase in PUFAs. Our results suggest a synergistic effect between EPA, vitamin D_3_, and the substitution of saturated fatty acids in anti-*P. salmonis* activity (Figure 3).

## 5. Conclusions

*M. gaditana* grown under controlled conditions can improve the synthesis of the anti-inflammatory eicosapentaenoic acid (EPA), which in turn can be used as a sustainable natural antimicrobial against *P. salmonis*. This approach confronts the issue of resistance to antibiotics, which impacts environmental and food safety risks. This natural therapy can reduce the cost of salmon meat production, mainly due to the reduction in the costs of smolts and antimicrobials, and can impact aquaculture sustainability as related to salmon farming. Our study provides evidence that *M. gaditana* is suitable for sustainable anti-*P. salmonis* feed additives for use in *S. salar* and a good candidate to test their efficacy in seawater challenge trials due to the presence of essential nutrients that play important roles in the fish immune system. *M. gaditana* provides new modalities to guarantee food safety and increase the nutritional quality of farmed Atlantic salmon.

## Figures and Tables

**Table 1:** Formulations, proximate, fatty acid, and vitamin D_3_ composition of the experimental diets.

Ingredient (%)	Control	Mg (1%)	Mg (10%)
Fish meal	28.00	28.00	28.00
Soybean (concentrate)	16.64	15.64	14.90
Whole wheat	14.00	14.00	
Wheat meal			7.00
Fish oil	19.70	19.70	18.40
Corn gluten	10.00	10.00	10.00
Viscera meal (chicken)	10.00	10.00	10.00
*Microchloropsis gaditana* (Mg)		1.00	10.00
Monocalcium phosphate	1.00	1.00	1.00
Mineral-vitamin premix	0.38	0.38	0.38
L-Lysine	0.21	0.21	0.27
DL-Methionine	0.07	0.07	0.08
Chemical composition			
Protein, %	45.76	45.96	44.38
Crude fat, %	19.54	19.61	19.66
Ash, %	9.84	10.03	11.08
Fiber, %	1.35	1.11	1.38
Moisture, %	6.68	6.28	6.54
Sodium %	0.38	0.40	1.08
Calcium %	1.10	1.11	1.21
Zinc, ppm	176	216	180
Vitamin D_3_ µg/100 g ^1^	15.40 ± 4.0	13.40 ± 3.50	12.80 ± 3.30
Fatty acid profile ^2^			
Myristic	6.21	6.23	6.32
Myristoleic	0.06	0.05	0.06
Palmitic	17.51	17.86	17.75
Palmitoleic	7.13	7.21	7.60
Margaric	0.39	0.40	0.29
Stearic	3.71	3.80	3.66
Elaidic	0.18	0.18	0.19
Oleic	12.43	12.56	12.25
Vaccenic	3.18	3.26	3.09
Linoleic	4.30	4.34	4.31
Linolenic	1.05	1.05	1.00
Gamma-Linolenic	0.21	0.21	0.22
Stearidonic	2.28	2.22	2.16
Arachidic	0.34	0.35	0.33
Gonodic	1.70	1.71	1.60
Homo-α-linolenic	0.08	0.08	0.09
Arachidonic	0.70	0.74	0.88
Eicosapentaenoic (EPA)	12.52	12.51	12.86
Behenoic	0.16	0.17	0.17
Erucic	0.34	0.34	0.33
C22:2n6	0.59	0.62	0.58
Adrenic	0.09	0.09	0.11
Docosapentaenoic (DPA)	1.62	1.62	1.53
Docosahexaenoic (DHA)	6.73	6.60	6.31
Lignoceric	0.10	0.09	0.10
Nervonic	0.44	0.46	0.45

^1^ Values based on the mean ± S.D. ^2^ Expressed as percent of total fatty acids.

**Table 2:** Chemical composition, vitamin D_3_, and fatty acid profile of Mg concentrate.

Moisture, %	4.44
Crude protein, %	36.54
Ash, %	21.26
Crude fat, %	4.18
Vitamin D_3_ µg/100g	<0.25
Calcium, %	4.11
Sodium, %	5.40
Zinc, ppm	171
Na/K ratio	5.40
Fatty Acid Profile (*as percent of total fatty acids*)	
Myristic	7.65
Myristoleic	0.16
C15:0	0.28
Palmitic	17.23
Palmitoleic	17.93
Stearic	0.33
Elaidic	0.19
Oleic	3.87
Vaccenic	0.37
Linoleic	3.17
Linolenic	0.17
Arachidonic (ARA)	3.92
Eicosapentaenoic (EPA)	26.73
Docosahexaenoic (DHA)	0.07
Lignoceric	0.20
n-3/n-6 PUFAs	3.56
ARA/EPA	0.15

**Table 3:** Growth performance indices of *Salmon salar* fed on *Microchloropsis gaditana* concentrate (Mg)-enriched diets for 49 days.

		Mg Levels (%)			
	Control (0.0)	1.0	*p* Value	10.0	*p* Value
Initial weight (g)	105.21 ± 0.88	105.02 ± 2.00	0.970	103.20 ± 0.55	0.173
Final weight (g)	208.19 ± 1.9	218.98 ± 6.13 *	0.023	219.56 ± 10.59 *	0.003
Final length (cm)	26.09 ± 0.10	26.06 ± 0.32		26.26 ± 0.65	
Weight gain (%)	102.99 ± 1.4	113.97 ± 8.03	0.053	116.36 ± 11.0 *	0.005
^1^ Specific growth rate (%/day)	1.39 ± 0.01	1.49 ± 0.09	0.099	1.59 ± 0.01 *	0.008
Condition factor	1.13 ± 0.01	1.15 ± 0.007	0.168	1.14 ± 0.01	0.485
^1^ Feed conversion ratio	1.11 ± 0.03	1.45 ± 0.24	0.145	0.93 ± 0.26	0.526
^1^ Survival rate (%)	97.33 ± 2.30	98.66 ± 2.30	0.716	98.66 ± 2.30	0.716
^1^ Feed Intake (%BW/day)	1.37 ± 0.03	1.57 ± 0.002	0.580	1.34 ± 0.44	0.986

* Significant difference from control at *p* < 0.05. ^1^ *n* = 3 (three replicate tanks of 25 fish), values are mean ± SD.

**Table 4:** Proximate, vitamin D_3_, and anti-inflammatory fatty acids composition of *Salmo salar* fillets fed on *Microchloropsis gaditana concentrate* (Mg)-enriched diets for 49 days, *n* = 3. * Significant difference from the control, *p* < 0.05, values are mean ± SD.

	Mg Levels (%)				
	Control (0.0)	1.0	*p* Value	10.0	*p* Value
Moisture, %	74.84 ± 0.17	75.33 ± 0.99	0.561	74.54 ± 0.39	0.799
Crude protein, %	19.07 ± 0.61	19.33 ± 0.80	0.875	19.21 ± 0.78	0.960
Ash, %	2.49 ± 0.18	2.57 ± 0.19	0.839	2.55 ± 0.20	0.920
Crude fat, %	4.22 ± 0.69	3.61 ± 0.24	0.403	4.30 ± 0.74	0.981
Vitamin D_3_ (µg/100 g)	2.58 ± 0.38	3.02 ± 0.68	0.776	5.32 ± 1.28 *	0.017
^1^ Fatty Acid Profile					
Myristic	4.42 ± 0.07	4.34 ± 0.04	0.334	4.48 ± 0.08	0.506
Palmitic	15.68 ± 0.16	15.15 ± 0.09	0.028	15.29 ± 0.28	0.850
Palmitoleic	6.00 ± 0.11	6.01 ± 0.01	0.998	6.26 ± 0.10 *	0.019
Stearic	4.03 ± 0.03	3.90 ± 0.04 *	0.007	3.80 ± 0.02 *	<0.000
Oleic	16.85 ± 1.01	15.99 ± 0.09	0.255	15.63 ± 0.46	0.104
Vaccenic	3.57 ± 0.03	3.61 ± 0.03	0.259	3.67 ± 0.01 *	0.008
Linoleic	6.71 ± 0.38	6.38 ± 0.08	0.250	6.15 ± 0.15	0.056
Linolenic	1.33 ± 0.09	1.28 ± 0.25	0.534	1.24 ± 0.03	0.153
C20:1n11	1.24 ± 0.06	1.34 ± 0.02	0.050	1.56 ± 0.01 *	<0.000
Arachidonic (ARA)	0.83 ± 0.02	0.83 ± 0.01	0.980	0.81 ± 0.03	0.544
Eicosapentaenoic (EPA)	7.56 ± 0.24	7.94 ± 0.09	0.221	8.33 ± 0.38 *	0.023
Erucic	0.27 ± 0.00	0.28 ± 0.00	0.382	0.29 ± 0.00 *	0.040
Cetoleic	1.62 ± 0.08	1.70 ± 0.02	0.270	1.91 ± 0.07 *	0.003
Docosapentaenoic (DPA)	2.94 ± 0.13	3.15 ± 0.07	0.093	3.31 ± 0.10 *	0.009
Docosahexanoic (DHA)	11.87 ± 0.89	12.85 ± 0.74	0.233	11.83 ± 0.39	0.997
∑ n-3/n-6	2.96 ± 0.25	3.22 ± 0.10	0.063	3.24 ± 0.13	0.053
∑ n-6/n-3	0.339 ± 0.02	0.309 ± 0.01	0.171	0.308 ± 0.01	0.154
EPA + DHA	19.43 ± 1.13	20.79 ± 0.65	0.217	20.16 ± 0.75	0.665
∑ n-6/EPA + DHA	0.446 ± 0.04	0.403 ± 0.01	0.216	0.407 ± 0.01	0.274
ARA/EPA	0.109 ± 0.001	0.104 ± 0.002	0.108	0.097 ± 0.002 *	0.001
Saturated	25.29 ± 0.19	24.50 ± 0.19 *	0.010	24.75 ± 0.28 *	0.045
Monounsaturated	31.63 ± 1.04	30.93 ± 0.13	0.437	31.46 ± 0.71	0.937
Polyunsaturated	34.16 ± 0.79	35.45 ± 0.45	0.104	34.80 ± 0.74	0.455

^1^ Expressed as percent of total fatty acids.

**Table 5:** *P. salmonis* antibacterial activity in sera from *S. salar* fed experimental diets, values are mean ± SD, *n* = 10. * Significantly different from control (*p* < 0.05), values are mean ± SD.

**Antibacterial Activity (%)**	**Day 0**	**Diet (Day 49)**				
		**Control**	**Mg 1 %**	***p* Value**	**Mg 10 %**	***p* Value**
	45.44 ± 19.83	51.01 ± 45.93	66.68 ± 48.10	0.128	84.59 ± 17.03 *	0.013

## Data Availability

Data are available on request from the authors.

## References

[1] SERNAPESCA (2022). Informe Sobre Uso de Antimicrobianos en la Salmonicultura Nacional. Subdirección de Acuicultura, Departamento de Salud Animal. http://www.sernapesca.cl/sites/default/files/informe_sobre_uso_de_antimicrobianos_en_la_salmonicultura_nacional_ano_2021.pdf.

[2] SERNAPESCA (2023). Informe con Antecedentes Sanitarios de Agua Dulce y Mar año, 1er Semestre 2023. https://www.sernapesca.cl/app/uploads/2023/12/Informe-Sanitario-1S-2023-Publicacion-002.pdf.

[3] Quiñones R.A., Fuentes M., Montes R.M., Soto D., León-Muñoz J. (2019). Environmental issues in Chilean salmon farming: A review. Rev. Aquac..

[4] Cabello F.C., Godfrey H.P., Ivanova L., Shah S.Q.A., Sørum H., Tomova A. (2020). Freshwater salmon aquaculture in Chile and transferable antimicrobial resistance. Environ. Microbiol..

[5] Love D.C., Fry J.P., Cabello F., Good C.M., Lunestad B.T. (2020). Veterinary drug use in United States net pen Salmon aquaculture: Implications for drug use policy. Aquaculture.

[6] Ramírez C., Gutiérrez M.S., Venegas L., Sapag C., Araya C., Caruffo M., López P., Reyes-Jara A., Toro M., González-Rocha G. (2022). Microbiota composition and susceptibility to florfenicol and oxytetracycline of bacterial isolates from mussels (*Mytilus* spp.) reared on different years and distance from salmon farms. Environ. Res..

[7] Lozano I., Díaz N.F., Muñoz  S., Riquelme C. (2018). Antibiotics in Chilean Aquaculture: A Review. Antibiot. Use Anim..

[8] Millanao A.R., Barrientos-Schaffeld C., Siegel-Tike C.D., Tomova A., Ivanova L., Godfrey H.P., Dölz H.J., Buschmann A.H., Cabello F.C., Millanao A.R. (2018). Antimicrobial resistance in Chile and The One Health paradigm: Dealing with threats to human and veterinary health resulting from antimicrobial use in salmon aquaculture and the clinic. Rev. Chil. Infectol..

[9] Lozano-Muñoz I., Wacyk J., Kretschmer C., Vásquez-Martínez Y., Cortez-San Martin M. (2021). Antimicrobial resistance in Chilean marine-farmed salmon: Improving food safety through One Health. One Health.

[10] Pisoschi A.M., Pop A., Georgescu C., Turcuş V., Olah N.K., Mathe E. (2018). An overview of natural antimicrobials role in food. Eur. J. Med. Chem..

[11] Cvitanich J.D., Garate n O., Smith C.E. (1991). The isolation of a rickettsia-like organism causing disease and mortality in Chilean salmonids and its confirmation by Koch’s postulate. J. Fish Dis..

[12] Sandoval R., Oliver C., Valdivia S., Valenzuela K., Haro R.E., Sánchez P., Olavarría V.H., Valenzuela P., Avendaño-Herrera R., Romero A. (2016). Resistance-nodulation-division efflux pump acrAB is modulated by florfenicol and contributes to drug resistance in the fish pathogen *Piscirickettsia salmonis*. FEMS Microbiol. Lett..

[13] Hossain A., Habibullah-Al-Mamun M., Nagano I., Masunaga S., Kitazawa D., Matsuda H. (2022). Antibiotics, antibiotic-resistant bacteria, and resistance genes in aquaculture: Risks, current concern, and future thinking. Environ. Sci. Pollut. Res..

[14] Mardones F.O., Paredes F., Medina M., Tello A., Valdivia V., Ibarra R., Correa J., Gelcich S. (2018). Identification of research gaps for highly infectious diseases in aquaculture: The case of the endemic *Piscirickettsia salmonis* in the Chilean salmon farming industry. Aquaculture.

[15] Contreras-Lynch S., Smith P., Olmos P., Loy M.E., Finnegan W., Miranda C.D. (2017). A Novel and Validated Protocol for Performing MIC Tests to Determine the Susceptibility of *Piscirickettsia salmonis* Isolates to Florfenicol and Oxytetracycline. Front. Microbiol..

[16] Cartes C., Isla A., Lagos F., Castro D., Muñoz M., Yañez A., Haussmann D., Figueroa J. (2016). Search and analysis of genes involved in antibiotic resistance in Chilean strains of *Piscirickettsia salmonis*. J. Fish Dis..

[17] Cabello F.C., Godfrey H.P. (2019). Salmon aquaculture, *Piscirickettsia salmonis* virulence, and one health: Dealing with harmful synergies between heavy antimicrobial use and piscine and human health. Aquaculture.

[18] Saavedra J., Hernandez N., Osses A., Castillo A., Cancino A., Grothusen H., Navas E., Henriquez P., Bohle H., Bustamante F. (2017). Prevalence, geographic distribution and phenotypic differences of *Piscirickettsia salmonis* EM-90-like isolates. J. Fish Dis..

[19] Henríquez P., Kaiser M., Bohle H., Bustos P., Mancilla M. (2016). Comprehensive antibiotic susceptibility profiling of Chilean *Piscirickettsia salmonis* field isolates. J. Fish Dis..

[20] Dawood M.A.O. (2021). Nutritional immunity of fish intestines: Important insights for sustainable aquaculture. Rev. Aquac..

[21] Arnemo M., Kavaliauskis A., Andresen A.M.S., Bou M., Berge G.M., Ruyter B., Gjøen T. (2017). Effects of dietary n-3 fatty acids on Toll-like receptor activation in primary leucocytes from *Atlantic salmon* (*Salmo salar*). Fish Physiol. Biochem..

[22] Miao L.H., Remø S.C., Espe M., Philip A.J.P., Hamre K., Fjelldal P.G., Skjærven K., Holen E., Vikeså V., Sissener N.H. (2022). Dietary plant oil supplemented with arachidonic acid and eicosapentaenoic acid affects the fatty acid composition and eicosanoid metabolism of *Atlantic salmon* (*Salmo salar* L.) during smoltification. Fish Shellfish Immunol..

[23] Andresen A.M.S., Lutfi E., Ruyter B., Berge G., Gjøen T. (2019). Interaction between dietary fatty acids and genotype on immune response in *Atlantic salmon* (*Salmo salar*) after vaccination: A transcriptome study. PLoS ONE.

[24] Roques S., Deborde C., Richard N., Skiba-Cassy S., Moing A., Fauconneau B. (2020). Metabolomics and fish nutrition: A review in the context of sustainable feed development. Rev. Aquac..

[25] Tocher D.R., Bell J.G., Dick J.R., Henderson R.J., McGhee F., Michell D., Morris P.C. (2000). Polyunsaturated fatty acid metabolism in *Atlantic salmon* (*Salmo salar*) undergoing parr-smolt transformation and the effects of dietary linseed and rapeseed oils. Fish Physiol. Biochem..

[26] Bou M., Berge G.M., Baeverfjord G., Sigholt T., Østbye T.-K., Romarheim O.H., Hatlen B., Leeuwis R., Venegas C., Ruyter B. (2017). Requirements of n-3 very long-chain PUFA in *Atlantic salmon* (*Salmo salar* L.): Effects of different dietary levels of EPA and DHA on fish performance and tissue composition and integrity. Br. J. Nutr..

[27] Sprague M., Dick J.R., Tocher D.R. (2016). Impact of sustainable feeds on omega-3 long-chain fatty acid levels in farmed *Atlantic salmon*, 2006–2015. Sci. Rep..

[28] Sissener N.H. (2018). Are we what we eat? Changes to the feed fatty acid composition of farmed salmon and its effects through the food chain. J. Exp. Biol..

[29] Neumann U., Derwenskus F., Gille A., Louis S., Schmid-Staiger U., Briviba K., Bischoff S.C. (2018). Bioavailability and safety of nutrients from the microalgae Chlorella vulgaris, *Nannochloropsis oceanica* and Phaeodactylum tricornutum in C57BL/6 mice. Nutrients.

[30] Lozano-Muñoz I., Muñoz S., Díaz N.F., Medina A., Bazaes J., Riquelme C. (2020). Nutritional Enhancement of Farmed Salmon Meat for Human Healthvia Non-GMO Nannochloropsis gaditana: Eicosapentaenoic Acid (EPA, 20:5n-3), Docosapentaenoic Acid (DPA, 22:5n-3) and Vitamin D3. Molecules.

[31] Sukarni, Sudjito, Hamidi N., Yanuhar U., Wardana I.N.G. (2014). Potential and properties of marine microalgae Nannochloropsis oculata as biomass fuel feedstock. Int. J. Energy Environ. Eng..

[32] Ljubic A., Jacobsen C., Holdt S.L., Jakobsen J. (2020). Microalgae *Nannochloropsis oceanica* as a future new natural source of vitamin D3. Food Chem..

[33] Mitra M., Patidar S.K., George B., Shah F., Mishra S. (2015). A euryhaline Nannochloropsis gaditana with potential for nutraceutical (EPA) and biodiesel production. Algal Res..

[34] Riveros K., Sepulveda C., Bazaes J., Marticorena P., Riquelme C., Acién G. (2018). Overall development of a bioprocess for the outdoor production of Nannochloropsis gaditana for aquaculture. Aquac. Res..

[35] Lock E.-J., Waagbø R., Bonga S.W., Flik G. (2010). The significance of vitamin D for fish: A review. Aquac. Nutr..

[36] Fernández I., Gavaia P., Darias M.J., Gisbert E., Yúfera M. (2018). Fat-Soluble Vitamins in Fish: A Transcriptional Tissue-Specific Crosstalk that Remains to be Unveiled and Characterized. Emerging Issues in Fish Larvae Research.

[37] Korf H., Decallonne B., Mathieu C. (2014). Vitamin D for infections. Curr. Opin. Endocrinol. Diabetes Obes..

[38] Guo C., Gombart A. (2014). The Antibiotic Effects of Vitamin D. Endocr. Metab. Immune Disord.-Drug Targets.

[39] Golpour A., Bereswill S., Heimesaat M.M. (2019). Antimicrobial and immune-modulatory effects of vitamin D provide promising antibiotics-independent approaches to tackle bacterial infections—Lessons learnt from a literature survey. Eur. J. Microbiol. Immunol..

[40] Analysis of Official Analytical Chemists International (2006). AOAC Official Method 954.02 Acid Hydrolysis, Baked Goods & Pet Food. https://www.aoac.org.

[41] Analysis of Official Analytical Chemists International (2000). AOAC Official Method 920.39 Crude Fat in Animal Feed. https://www.aoac.org.

[42] Analysis of Official Analytical Chemists International (2012). AOAC Official Method 996.06 Analysis of Methyl esters by Capillary GLC. https://www.aoac.org.

[43] Analysis of Official Analytical Chemists International (1995). Protein (crude) in animal feed and pet food 984.13. Off. Methods Anal. Off. Anal. Chem. Int..

[44] Analysis of Official Analytical Chemists International AOAC Official Method 942.05 Determinaton of Ash in Animal feed. 2012, 1392–1397. https://www.aoac.org.

[45] Analysis of Official Analytical Chemists International AOAC Official Method 934.01 Loss on Drying (Moisture) at 95–100 °C for Feeds 2000. https://www.aoac.org.

[46] (2009). Foodstuffs—Determination of Vitamin D by High Performance Liquid Chromatography. Measurement of Cholecalciferol (D3) or Ergocalciferol (D2).

[47] Kilkenny C., Browne W.J., Cuthill I.C., Emerson M., Altman D.G. (2010). Improving Bioscience Research Reporting: The ARRIVE Guidelines for Reporting Animal Research. PLoS Biol..

[48] Reid D.P., Szanto A., Glebe B., Danzmann R.G., Ferguson M.M. (2005). QTL for body weight and condition factor in *Atlantic salmon* (*Salmo salar*): Comparative analysis with rainbow trout (*Oncorhynchus mykiss*) and Arctic charr (*Salvelinus alpinus*). Heredity.

[49] Topic Popovic N., Strunjak-Perovic I., Coz-Rakovac R., Barisic J., Jadan M., Persin Berakovic A., Sauerborn Klobucar R. (2012). Tricaine methane-sulfonate (MS-222) application in fish anaesthesia. J. Appl. Ichthyol..

[50] (2010). Directiva 210/63. Directiva 2010/63/UE del parlamento europeo y del consejo de 22 de septiembre de 2010 relativa a la protección de los animales utilizados para fines científicos (Texto pertinente a efectos del EEE). D. Of. Comunidades Eur..

[51] AOAC AOAC Official Method 948.15, Fat (crude) in Seafood. Acid Hydrolysis Method 1948. https://www.aoac.org.

[52] AOAC AOAC Official Method 938.08, Ash of Seafood 1938. https://www.aoac.org.

[53] Yañez A., Valenzuela K., Silva H., Retamales J., Romero A., Enriquez R., Figueroa J., Claude A., Gonzalez J., Avendaño-Herrera R. (2012). Broth medium for the successful culture of the fish pathogen *Piscirickettsia salmonis*. Dis. Aquat. Organ..

[54] Yañez A.J., Silva H., Valenzuela K., Pontigo J.P., Godoy M., Troncoso J., Romero A., Figueroa J., Carcamo J.G., Avendaño-Herrera R. (2013). Two novel blood-free solid media for the culture of the salmonid pathogen *Piscirickettsia salmonis*. J Fish Dis.

[55] Dang V.T., Speck P., Doroudi M., Smith B., Benkendorff K. (2011). Variation in the antiviral and antibacterial activity of abalone *Haliotis laevigata*, *H. rubra* and their hybrid in South Australia. Aquaculture.

[56] Alves S.P., Mendonça S.H., Silva J.L., Bessa R.J.B. (2018). *Nannochloropsis oceanica*, a novel natural source of rumen-protected eicosapentaenoic acid (EPA) for ruminants. Sci. Rep..

[57] Gong Y., Sørensen S.L., Dahle D., Nadanasabesan N., Dias J., Valente L.M.P., Sørensen M., Kiron V. (2020). Approaches to improve utilization of *Nannochloropsis oceanica* in plant-based feeds for *Atlantic salmon*. Aquaculture.

[58] Nagappan S., Das P., AbdulQuadir M., Thaher M., Khan S., Mahata C., Al-Jabri H., Vatland A.K., Kumar G. (2021). Potential of microalgae as a sustainable feed ingredient for aquaculture. J. Biotechnol..

[59] Sørensen S.L., Ghirmay A., Gong Y., Dahle D., Vasanth G., Sørensen M., Kiron V. (2021). Growth, Chemical Composition, Histology and Antioxidant Genes of Atlantic Salmon (*Salmo salar*) Fed Whole or Pre-Processed *Nannochloropsis oceanica* and *Tetraselmis* sp.. Fishes.

[60] Hu W., Zhang L., Li M.X., Shen J., Liu X.D., Xiao Z.G., Wu D.L., Ho I.H.T., Wu J.C.Y., Cheung C.K.Y. (2019). Vitamin D3 activates the autolysosomal degradation function against *Helicobacter pylori* through the PDIA3 receptor in gastric epithelial cells. Autophagy.

[61] Almoudi M.M.M., Hussein A.S., Abu Hassan M.I., Al Talib H., Khan H.B.S.G., Nazli S.A.B., Effandy N.A.E.B. (2021). The antibacterial effects of vitamin D3 against mutans streptococci: An in vitro study. Eur. Oral Res..

[62] Liu J., Shao R., Lan Y., Liao X., Zhang J., Mai K., Ai Q., Wan M. (2021). Vitamin D3 protects turbot (*Scophthalmus maximus* L.) from bacterial infection. Fish Shellfish Immunol..

[63] Saini R.K., Prasad P., Sreedhar R.V., Akhilender Naidu K., Shang X., Keum Y.-S. (2021). Omega- 3 polyunsaturated fatty acids (PUFAs): Emerging plant and microbial sources, oxidative stability, bioavailability, and health benefits—A review. Antioxidants.

[64] Bastías J.M., Balladares P., Acuña S., Quevedo R., Muñoz O. (2017). Determining the effect of different cooking methods on the nutritional composition of salmon (*Salmo salar*) and chilean jack mackerel (*Trachurus murphyi*) fillets. PLoS ONE.

[65] Toshkova-Yotova T., Georgieva A., Iliev I., Alexandrov S., Ivanova A., Pilarski P., Toshkova R. (2022). Antitumor and antimicrobial activity of fatty acids from green microalga *Coelastrella* sp. BGV. S. Afr. J. Bot..

[66] Domb A.J., Kunduru K.R., Farah S. (2019). Antimicrobial Materials for Biomedical Applications.

[67] Ibrahim D., Abd El-Hamid M.I., Al-Zaban M.I., ElHady M., El-Azzouny M.M., ElFeky T.M., Al Sadik G.M., Samy O.M., Hamed T.A., Albalwe F.M. (2022). Impacts of Fortifying Nile Tilapia (*Oreochromis niloticus*) Diet with Different Strains of Microalgae on Its Performance, Fillet Quality and Disease Resistance to *Aeromonas hydrophila* Considering the Interplay between Antioxidant and Inflammatory Response. Antioxidants.

[68] Guzmán F., Wong G., Román T., Cárdenas C., Alvárez C., Schmitt P., Albericio F., Rojas V. (2019). Identification of Antimicrobial Peptides from the *Microalgae Tetraselmis suecica* (Kylin) Butcher and Bactericidal Activity Improvement. Mar. Drugs.

[69] Bahi A., Ramos-Vega A., Angulo C., Monreal-Escalante E., Guardiola F.A. (2023). Microalgae with immunomodulatory effects on fish. Rev. Aquac..

[70] Tülay Çağatay I., Özbaş M., Yilmaz H.E., Ali N. (2021). Determination of Antibacterial Effect of Nannochloropsis oculata Against Some Rainbow Trout Pathogens. Nat. Eng. Sci..

[71] Putra Y., Mustikasari I., Pangestuti R., Rahmadi P., Siahaan E.A. (2022). Fatty acid profiles and biological activity of Nannochloropsis oculata and Isochrysis galbana, clone t-ISO. IOP Conf. Ser. Earth Environ. Sci..

[72] Martinez-Rubio L., Morais S., Evensen Ø., Wadsworth S., Vecino J.G., Ruohonen K., Bell J.G., Tocher D.R. (2013). Effect of functional feeds on fatty acid and eicosanoid metabolism in liver and head kidney of *Atlantic salmon* (*Salmo salar* L.) with experimentally induced Heart and Skeletal Muscle Inflammation. Fish Shellfish Immunol..

[73] Fritsche K.L. (2015). The Science of Fatty Acids and Inflammation. Adv. Nutr..

[74] Lamon-Fava S., So J., Mischoulon D., Ziegler T.R., Dunlop B.W., Kinkead B., Schettler P.J., Nierenberg A.A., Felger J.C., Maddipati K.R. (2021). Dose- and time-dependent increase in circulating anti-inflammatory and pro-resolving lipid mediators following eicosapentaenoic acid supplementation in patients with major depressive disorder and chronic inflammation. Prostaglandins Leukot. Essent. Fatty Acids.

[75] Pussinen P.J., Kopra E., Pietiäinen M., Lehto M., Zaric S., Paju S., Salminen A. (2022). Periodontitis and cardiometabolic disorders: The role of lipopolysaccharide and endotoxemia. Periodontology 2000.

[76] Ortiz-Severín J., Travisany D., Maass A., Cambiazo V., Chávez F.P. (2020). Global Proteomic Profiling of *Piscirickettsia salmonis* and Salmon Macrophage-Like Cells during Intracellular Infection. Microorganisms.

[77] Marín-Palma D., Taborda N.A., Urcuqui-Inchima S., Hernandez J.C. (2017). Inflamación y respuesta inmune innata: Participación de las lipoproteínas de alta densidad. Iatreia.

[78] Panpetch W., Sawaswong V., Chanchaem P., Ondee T., Dang C.P., Payungporn S., Tumwasorn S., Leelahavanichkul A. (2020). Candida Administration Worsens Cecal Ligation and Puncture-Induced Sepsis in Obese Mice Through Gut Dysbiosis Enhanced Systemic Inflammation, Impact of Pathogen-Associated Molecules From Gut Translocation and Saturated Fatty Acid. Front. Immunol..

[79] Keller R., Fischer W., Keist R., Bassetti S. (1992). Macrophage response to bacteria: Induction of marked secretory and cellular activities by lipoteichoic acids. Infect. Immun..

[80] Namgaladze D., Brüne B. (2016). Macrophage fatty acid oxidation and its roles in macrophage polarization and fatty acid-induced inflammation. Biochim. Biophys. Acta BBA Mol. Cell Biol. Lipids.

